# ADAR1 plays a protective role in proximal tubular cells under high glucose conditions by attenuating the PI3K/AKT/mTOR signaling pathway

**DOI:** 10.1515/med-2024-1037

**Published:** 2024-10-10

**Authors:** Ying Wang, Jiang Chang, Fa Wang, Lianying Lai, ShiXu Yang, Yueying Fu, Xingtian Ma, Chuan Yun

**Affiliations:** Department of General Practice, The First Affiliated Hospital of Hainan Medical University, Haikou, 570102, Hainan, China; Department of Hepatobiliary Surgery, The First Affiliated Hospital of Hainan Medical University, Haikou, 570102, Hainan, China; Department of Anesthesiology, Ningxia Medical University, Yinchuan, 750004, Ningxia Hui Autonomous Region, China; Department of Anesthesiology, Bayannur Hospital, Bayannur, Inner Mongolia Autonomous Region, 015000, China; Department of Nephrology, Hainan Medical University, Haikou, 570102, Hainan, China; Department of Endocrinology, The First Affiliated Hospital of Hainan Medical University, Haikou, 570102, Hainan, China

**Keywords:** ADAR1, diabetic kidney disease, endoplasmic reticulum stress, oxidative stress

## Abstract

**Background:**

Adenosine deaminases acting on RNA 1 (ADAR1), an RNA editing enzyme, holds a role in cancer, inflammation, and immunity. However, its specific function in the nephropathy and high-glucose-induced human renal tubular epithelial cells (HK-2) injury in diabetic db/db mice is not clear.

**Methods:**

This study explored the expression characteristics of ADAR1 in proximal renal tubular cells of diabetic db/db mice, examining its function in the mechanism of high-glucose-induced HK-2 cell injury. Furthermore, it elucidated the molecular mechanism underlying the protective effect of ADAR1, the regulation of phosphatidylinositol 3-kinase (PI3K)/protein kinase B (PKB/Akt)/mammalian target of the rapamycin (mTOR) signaling. We observed a decrease in ADAR1 expression in proximal tubular cells of diabetic db/db mice, accompanied by an increase in the expression of inflammation-related markers (PI3K/AKT/mTOR).

**Results:**

We constructed and validated ADAR1-overexpression plasmids and used an ADAR1 inhibitor (8-azaadenosine) to carry out cell experiments. The upregulation of ADAR1 expression alleviated high-glucose-induced endoplasmic reticulum stress, reduced HK-2 cell apoptosis, and reduced the expression of inflammation-related indicators (PI3K/AKT/mTOR).

**Conclusion:**

Taken together, the pivotal roles of ADAR1 in the progression of proximal renal tubulopathy and the mechanism of high-glucose-induced HK-2 injury in diabetic db/db mice suggest that ADAR1 may be a potential key factor in slowing the progression of diabetic kidney disease.

## 1 Introduction

Diabetic kidney disease (DKD) is a common complication in the end stage of long-term disease in diabetic patients. In the progression of DKD, the dysfunction associated with both glomerulopathy and renal tubulopathy play a pivotal role [1]. In renal tubulopathy, proximal tubular cell function is highly susceptible to diabetic disorders, which can trigger a cascade of deleterious effects including energy imbalances, oxidative stress, endoplasmic reticulum (ER) stress, and apoptosis [2]. Renal tubular epithelial cell injury is intimately linked to the development of DKD [3]. Prolonged exposure to high-glucose levels stimulates a cascade of deleterious effects, including the overproduction of reactive oxygen species, which instigates oxidative stress and triggers an inflammatory cascade. This cascade, in turn, accelerates apoptotic processes in renal tubular epithelial cells, culminating in renal tubular damage [4,5].

Adenosine deaminases acting on RNA 1 (ADAR1) is an RNA editing enzyme and it mainly catalyzes the conversion of adenosine to inosine in coding and noncoding RNA transcripts (A-to-I RNA editing). This process destabilizes double-stranded RNA (dsRNA) [6]. ADAR1 exists in two subtypes, p110 and p150. The p110 subtype is mainly expressed in the nucleus, and is associated with a number of diseases. The p150 subtype is mainly expressed in the cytoplasm, with a small amount expressed in the nucleus [7]. ADAR1 p110 and p150 subtypes differentiate from each other in molecular mass, intracellular localization, regulatory mechanisms governing, functional focus, and potential disease associations. These disparities underscore their diverse roles in the fine regulation and response to a broad spectrum of physiological and pathophysiological states within cellular environments [8]. 8-Azaadenosine blocks ADAR1 expression and inhibits thyroid cancer cell proliferation, 3D growth, invasion, and migration. In the realm of cancer investigations, ADAR1 p110 is mainly expressed in the nucleus of gastric cancer (GC) cells. It regulates β-catenin signaling and epithelial to mesenchymal transition signaling, integral to metastatic progression [9]. In non-cancer studies, ADAR1 p110 has demonstrated a role in facilitating enterovirus replication by suppressing the deaminase domain within the PKR pathway [10]. ADAR1 p150 is exclusively responsible for ADAR1 function in germinal center reactions, and the p110 isoform cannot serve the same role as p150 [11]. ADAR1 p150 is involved in antiviral and immune regulation through editing of viral dsRNA [12]. ADAR1-mediated adenosine–inosine RNA editing is an important posttranscriptional event that suppresses cellular dsRNA-mediated innate immune interferon responses and thereby functioning as a key mechanism of genetic variation associated with common inflammatory diseases [13]. The overexpression of ADAR1 inhibits the expansion of white adipose tissue in high-fat diet-induced obese mice, thereby improving metabolic phenotypes such as insulin sensitivity and glucose tolerance [14]. Research has further illuminated that a lack of ADAR1 exerts a protective effect against obesity and insulin resistance induced by high-fat diets in mice models [15]. In diabetes, it is believed that the phosphatidyl inositol 3-kinase (PI3K)/protein kinase B (PKB/Akt)/mammalian target of the rapamycin (mTOR) signaling is widely acknowledged as a fundamental pathway regulating cell proliferation, differentiation, and apoptosis [16]. In human umbilical vein endothelial cells, ADAR1 regulates cell proliferation and permeability by activating the PI3K-Akt pathway [17]. In the context of a GC study, ADAR1 has been shown to exert a regulatory influence on the activity of the mTOR signaling pathway. When ADAR1 expression is suppressed, there is a significant decrease in the phosphorylation levels of mTOR, p70S6K, and the S6 ribosomal protein [18].

The roles of ADAR1 are of remarkable significance in diabetes, autologous diseases, and cancer [7]. However, the mechanism through which ADAR1 operates in DKD is unclear. This study aimed to clarify the critical role of ADAR1 in the progression of proximal renal tubulopathy and the mechanism of high-glucose-induced HK-2 injury in diabetic db/db mice. By measuring ADAR1 expression in proximal renal tubulopathy in diabetic db/db mice, the study illuminated that ADAR1 can alleviate high-glucose-induced apoptosis, oxidative stress, and ER stress in HK-2 cells through PI3K/AKT/mTOR. This study was conducted using spontaneous type 2 diabetic db/db mice and high-glucose-induced human proximal tubular epithelial (HK-2) cells.

## 2 Materials and methods

### 2.1 Experiment animals and cells

#### 2.1.1 Animals

Male diabetic db/db mice and db/m mice (SPF) were purchased from Cavens Laboratory Animal Co., Ltd (Changzhou, China; License key: SCXK (Su) 2021-0013). At age of 6–8 weeks, the weight reached 16–18 g. Mice breeding and experiment were both performed at Laboratory Animal Center, Hainan Medical College. All experiments strictly followed the Guide for the Care and Use of Laboratory Animals published by the US National Institute of Health and gained approval from the Ethics Committee of Hainan Medical College (HYLL-2021-296). Mice were bred in temperature-controlled (25℃) room on a 12 h light/dark schedule. The experiment was conducted for 6 weeks and the mice were divided into two groups: the db/m group (*n* = 5) and the db/db group (*n* = 5). Weight, water, and food intake of mice were measured every 6 days. Yuwell blood glucose meter was used to detect the fasting blood glucose (FBG) every 2 weeks [19]. Mice euthanasia was performed by intraperitoneal injection of pentobarbital sodium (50 mg/kg) (Tc-P8411, Merck).

#### 2.1.2 Cells

Human renal tubular epithelial cells were purchased from Zhong Qiao Xin Zhou Biotechnology Co., Ltd, (Shanghai, China) (ZQ0313, HK-2) and cultured with specific medium for HK-2 cells, at 37℃, 5% CO_2_ according to the instruction. HK-2 cells were exposed to normal glucose (5.6 mmol/L) or high sugar (30 mmol/L), then added ADAR1 inhibitors (8-azaadenosine) or transfection overexpression plasmid into cells. Experimental groups were as follows: control (5.6 mmol/L normal glucose), HG (30 mmol/L glucose), HG + 8-azaadenosine (30 mmol/L glucose + 2 μM), and HG + OE-ADAR1.

### 2.2 Cell viability assay-cell counting kit-8 (CCK8)

About 100 µL stably transferred cell suspension was added to each hole of a 96-well plate at a density of 1 × 10^4^ cells/well. After cultured at 37℃ in an incubator for 24 h, varied concentrations of ADAR1 inhibitor (8-azaadenosine, 1, 2, 4, 6, 8, 10, and 20 μg/mL) (HY-115686, MCE), PI3K inhibitor (LY294002, 5, 10, 15, 20, 40, 80, and 100 μM) (HY-10108, MCE), and mTOR in inhibitor (rapamycin, 5, 10, 15, 20, 40, 80, and 100 μM) (HY-10219, MCE) were added in different wells. After culturing at 37℃ for 24 h, 10 µL of CCK8 (Dojindo, Shanghai, China) reagent was added to each well. Then after culturing at 37℃ for 3 h, the absorbance of each well was measured at 450 nm using microplate reader (Rayto, China).

### 2.3 Measurement of superoxide dismutase (SOD) activity

SOD activity in HK-2 cell was measured using CuZn/Mn-SOD activity assay kit (S0103, Beyotime, China). Briefly, cell homogenate was collected using PBS buffer in ice bath and then centrifuged at 4℃. The 20 μL supernatant of the sample was added to the reagents according to the kit instructions and absorbance was read at 450 nm after 30 min incubation.

### 2.4 Malondialdehyde (MDA)

The level of MDA was measured using MDA assay kits (S0131S, Beyotime, China). Briefly, the cells were lysed in a lysis buffer in ice bath. Then 100 μL supernatant of the sample was added to the reagents according to the kit instructions. The mixture is then heated in boiling water bath for 15 min and centrifuged at 1,000*g* for 10 min. Subsequently, 200 μL of the supernatant was added to 96-well plate, after which the absorbance was read at 532 nm.

### 2.5 ER-Tracker Red

ER-Tracker Red Kit (C1041, Beyotime, China) was used to stain the ER according to the kit instructions. After cell treatment and incubation, the cell medium was removed and the cells were washed with PBS buffer. Then dilute the ER Tracker Red solution in a 1:1,000 ratio and obtain the ER Tracker Red working liquid. Cells were incubated in pre-warmed ER-tracker working liquid at 37℃ for 20 min. After removing ER-tracker dye solution, the cells were washed with cell medium twice, and observed using a fluorescence microscope.

### 2.6 TUNEL staining

Cells were fixed in 4% paraformaldehyde for 30 min, and then incubated with 0.3% Triton X-100 in PBS for 5 min at room temperature, succeeded by three washes with PBS. Labeling was achieved after applying 50 μL of labeling solution, with a 60 min incubation at 37°C in the dark. Post-PBS washing, nuclei were stained using DAPI. The apoptosis rate was subsequently quantified, and photomicrographs were captured under fluorescence microscopy to document the findings (the red fluorescent label indicates apoptotic cells).

### 2.7 Transfection

Overexpression plasmids of ADAR1 was constructed by Sangon Biotech Co., Ltd (Shanghai, China) (ID: 103, cloning vector: pcDNA3.1(+)). When 70–80% of cell fusion is achieved, Lipo8000^TM^ (C0533-1.5 mL, Beyotime) was used to transfect the plasmids into HK-2 cells. After incubation for 48 h, HK-2 cells were collected to detect the efficiency of transfection by reverse transcription-polymerase chain reaction (RT-PCR).

### 2.8 Cell western blot assay

Protein was extracted from cells with Whole Cell Lysis Assay Kit (KeyGEN BioTECH, China) containing lysis buffer, protease inhibitors, phosphatase inhibitors, and 100 mM phenylmethylsulfonyl fluoride. BCA Protein Assay Kit (LEAGENE, China) was used to detect the protein concentration. About 30 ng of protein of each sample was then separated with 11% SDS-PAGE for immunoblot and transferred to polyvinylidene fluoride (PVDF) membrane. After 2 h of blocking in 5% skim milk at room temperature, the membranes were incubated with primary antibodies at 4℃ overnight. Primary antibodies and corresponding dilution rate are as follows: ADAR1 (1:100, sc-271854; SANTA CRUZ, USA), PI3K (1:1,000, AF6241; Affinity, China), phospho-PI3K (1:500, AF3241; Affinity, China), phospho-AKT (1:500, 80455-1-RR; Proteintech, China), AKT (1:1,000, 10176-2-AP; Proteintech, China), phospho-mTOR (1:500, 67778-1-Ig; Proteintech, China), mTOR (1:1,000, 28273-1-AP; Proteintech, China), cyclin D1 (1:1,000, 26939-1-AP; Proteintech, China), Ki-67 (1:1,000, 28074-1-AP; Proteintech, China), and β-actin (1:2,000, 20536-1-AP; Proteintech, China). Then the membranes were washed with Tris-buffered saline with Tween 20 (TBST) (T1082, Solarbio) three times and incubated with HRP-conjugated goat-anti-rabbit IgG (ZB-2301, ZSGB-Bio) or HRP-conjugated goat-anti-mouse IgG (ZB-2305, ZSGB-Bio) for 1 h at room temperature. The bands were visualized by enhanced chemiluminescence (ECL) hypersensitive luminescence solution.

### 2.9 Kidney tissue western blot assay

Tissue homogenate was collected, lysed with 200 μL RIPA buffer for 30 min, and then centrifuged at 12,000 rpm for 15 min. The supernatant was used to detect the protein concentration of each sample. About 30 ng of protein of each sample was then separated with 11% SDS-PAGE for immunoblot and transferred to PVDF membrane. After 2 h of blocking in 5% skim milk at room temperature, the membranes were incubated with primary antibodies at 4℃ overnight. Primary antibodies and corresponding dilution rate are as follows: ADAR1 (1:100, sc-271854; SANTA CRUZ, USA), phospho-PI3K (1:500, AF3241; Affinity, China), phospho-AKT (1:500, 80455-1-RR; Proteintech, China), and phospho-mTOR (1:500, 67778-1-Ig; Proteintech, China). Then the membranes were washed with TBST three times and incubated with HRP-conjugated goat-anti-rabbit IgG or HRP-conjugated goat-anti-mice IgG for 1 h at room temperature. The bands were visualized by ECL hypersensitive luminescence solution.

### 2.10 Immunohistochemistry (IHC)

After paraffin sections were deparaffinized and rehydrated, antigen retrieval was performed using EDTA antigen retrieval solution (PH8.0, ZSGB-BIO, China) in high pressure for 3 min. The cooled paraffin sections were incubated in 3% hydrogen peroxide at room temperature for 15 min and then incubated in bovine serum albumin at 37℃ for 30 min. Followed by incubating with primary antibodies: ADAR1 (1:50, sc-271854; SANTA CRUZ, USA), phospho-PI3K (1:100, AF3241; Affinity, China), phospho-AKT (1:100, 80455-1-RR; Proteintech, China), and phospho-mTOR (1:100, 67778-1-Ig; Proteintech, China) at 4℃ overnight, the paraffin sections were incubated with secondary antibody (PV6000, ZSGB-BIO, China) at 37℃ for 20 min. Immunohistochemical staining was then carried out by DAB (ZLI-9019, ZSGB-Bio), redyed by hematoxylin, and evaluated by light microscopy.

### 2.11 Oral glucose tolerance test (OGTT)

OGTT was performed after the mice fasted for 10 h. The dose of glucose used was 2 g/kg. Blood glucose was measured at 0, 30, 60, 90, and 120 min.

### 2.12 Hematoxylin and eosin (H&E) staining

Tissue samples were subjected to fixation in a 4% paraformaldehyde solution maintained at 4°C for a 24 h duration. Subsequently, paraffin embedding was performed, yielding 5 µm thick sections. These sections underwent staining procedures with 0.5% hematoxylin and 0.5% eosin solution (G1120; Solarbio) for 5 min at a temperature of 37°C.

### 2.13 Masson’s staining

Masson’s trichrome staining kit (BA4079B, Baso, China) was used to stain the tissue for fibrosis analysis. About 5 µm thick paraffin sections are routinely dewaxed in water and the Weigert hematoxylin A and B solutions are removed and mixed in a 1:1 ratio, which are then used to completely cover the tissue for 5 min. Sections are then stained with fuchsin acidic hematoxylin solution for 3 min, and again stained with phosphomolybdic acid solution for 5 min before being stained with aniline blue solution for 5 min. The tissue is then soaked in 0.2% acetic acid solution, routinely dehydrated and cleared, and mounted with neutral resin.

### 2.14 Biochemical indexes

Serum specimens were separated from the collected blood according to manufacturer’s protocols. The levels of blood creatinine (Cr) (C011-2-1, Nanjing Jiancheng Bioengineering Institute) and blood urea nitrogen (BUN) (C013-2-1, Nanjing Jiancheng Bioengineering Institute) were detected.

### 2.15 Determination of serum insulin

According to manufacturer’s protocol, mouse serum insulin concentration was examined using a mouse insulin ELISA kit (JYM0351Mo; Wuhan Jiyinmei Biotechnology Co., Ltd). The absorbance value was examined at 450 nm using a microplate reader.

### 2.16 Immunofluorescence staining

The paraffin sections were dewaxed for 30 min, repaired under high pressure, and washed three times with PBS. Permeabilize them with 0.1% Triton X-100 at 37°C for 20 min. Block the cells with goat serum at 37°C for 20 min. Add the primary antibody and incubate them at 4°C overnight in a humidified box. The antibodies used are ADAR1 (1:40, sc-271854; SANTA CRUZ, USA), phospho-PI3K (1:100, AF3241; Affinity, China), phospho-AKT (1:100, 80455-1-RR; Proteintech, China), and phospho-mTOR (1:100, 67778-1-Ig; Proteintech, China).

The next day, the samples were rewarmed and washed with PBS three times. The secondary antibody Alexa Fluor^®^ 488 Conjugate (1:100, #ZF-0511; ZSGB-BIO) as well as Alexa Fluor^®^ 594 Conjugate (1:100, #ZF-0513; ZSGB-BIO) was then added and incubated in the dark at 37°C for 60 min. 4',6-diamidino-2-phenylindole was added, the slide was sealed, and the samples were observed under a fluorescence microscope.

### 2.17 RT-PCR analysis

Total RNA was extracted by TRIzol^®^ (Thermo Fisher Scientific, Waltham, MA, USA).

The cDNA was obtained using RevertAid First Strand cDNA Synthesis Kit (Thermo Fisher Scientific, Inc.). The primer sequences used were as follows:

ADAR1-F: 5′-CTGAGACCAAAAGAAACGCAGA-3′

ADAR1-R: 5’-GCCATTGTAATGAACAGGTGGTT-3′

GAPDH-F: 5’-TCCACCACCCTGTTGCTGTA-3′

GAPDH-R: 5’-ACCACAGTCCATGCCATCAC-3′.

mRNA levels were assessed using the PowerUp™ SYBR™ Green Master Mix (Applied Biosystems; Thermo Fisher Scientific, Inc.). Samples were denatured at 95°C for 30 s, followed by 40 cycles of 95°C for 5 s; 60°C for 40 s; 95°C for 15 s; 60 s at 60°C, 95°C for 15 s. GAPDH mRNA levels were assessed as an internal control. Relative gene expression levels were normalized using the 2^−ΔΔCq^ method.

### 2.18 Statistical analysis

SPSS 22.0 was used for statistical analysis, and the data were expressed as mean ± standard deviation. Analysis of variance was used to compare the mean of samples in multiple groups, and LSD-*t* test was used to compare the mean of samples in pairs within groups, with the test level *α* = 0.05.


**Ethical approval:** This study was performed in line with the principles of the Declaration of Helsinki. Approval was granted by the Ethics Committee of Hainan Medical College (HYLL-2021-296).

## 3 Results

### 3.1 Expression characteristics of ADAR1 in proximal renal tubular cells of db/db mice with spontaneous type 2 diabetes mellitus

**Figure 1:**
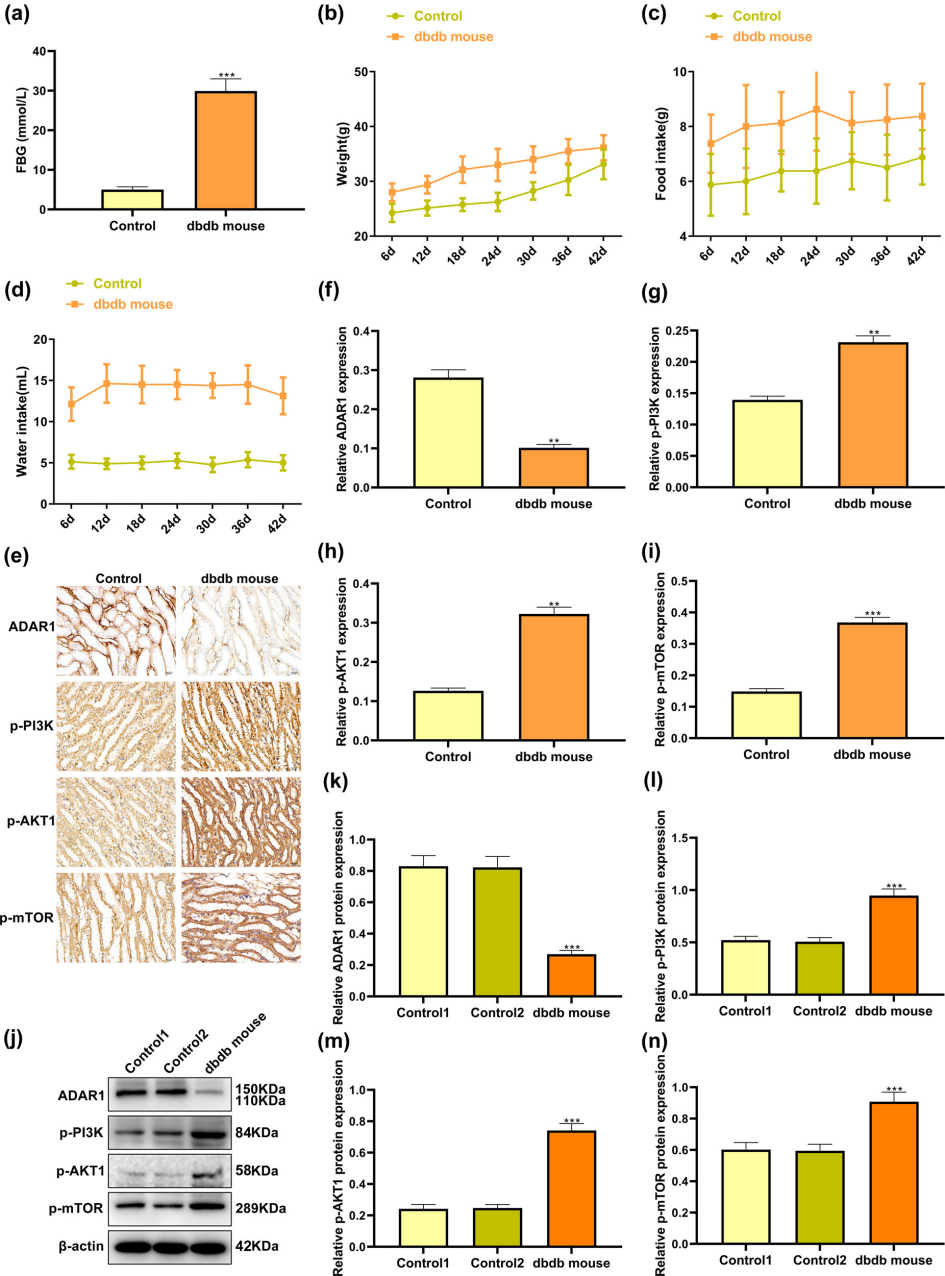
Detect the expression characteristics of ADAR1 in proximal renal tubular cells of db/db mice with spontaneous type 2 diabetes mellitus. (a) Detect FBG, (b) weight, (c) food intake, and (d) water intake of db/db mice with spontaneous type 2 diabetes mellitus and normal db/m mice. (e)–(i) IHC assays were presented for detecting the expression of (f) ADAR1, (g) p-PI3K, (h) p-AKT1, and (i) p-mTOR. (j)–(n) Western blot was presented for detecting the expression of (k) ADAR1, (l) p-PI3K, (m) p-AKT1, and (n) p-mTOR. Bar, 20 μm. *p < 0.05; **p < 0.01; ***p < 0.001.

**Figure A1:**
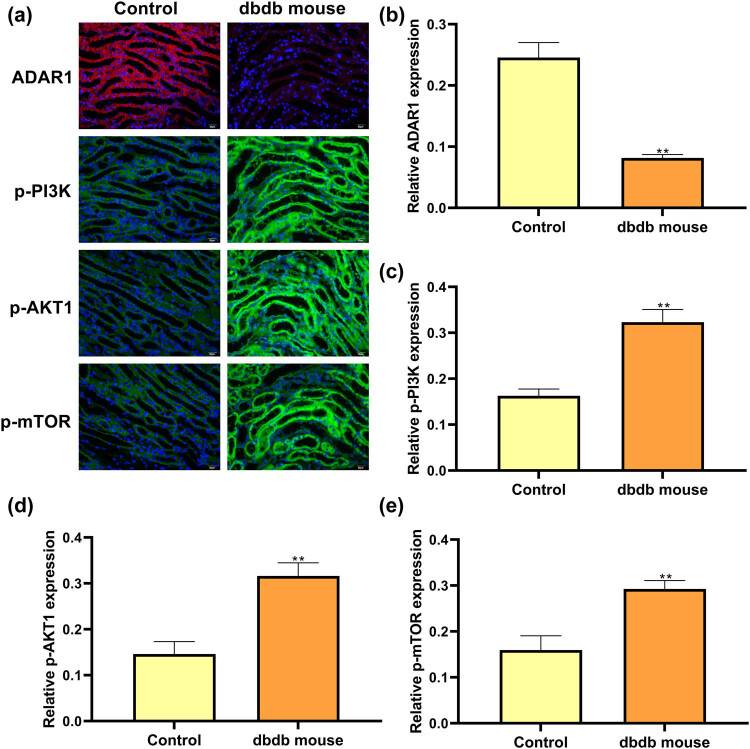
Detect the expression characteristics of ADAR1 in proximal renal tubular cells of db/db mice with spontaneous type 2 diabetes mellitus.(a) Immunofluorescence assays were presented for detecting the expression of (b) ADAR1, (c) p-PI3K, (d) p-AKT1, and (e) p-mTOR. Bar, 20 μm. **p < 0.01.

The FBG, body weight, food, and water intake of db/db mice with spontaneous type 2 diabetes were significantly higher than those of mice in the normal control group (Figure 1a–d). OGTT results showed that the blood glucose of db/db mice group was significantly higher than that of control group (Figure A1g). In addition, the levels of blood Cr, insulin, and BUN in db/db mice were also increased (Figure A1d–f). H&E staining showed that db/db mice group had dilation of renal tubules and a marked disorganization of tubular epithelial cells compared with the control group (Figure A1a). In the comparative analysis utilizing Masson’s staining, db/db mice had more blue-stained collagen fibers infiltrating the proximal tubule interstitium than the control (Figure A1b and c).

**Figure A2:**
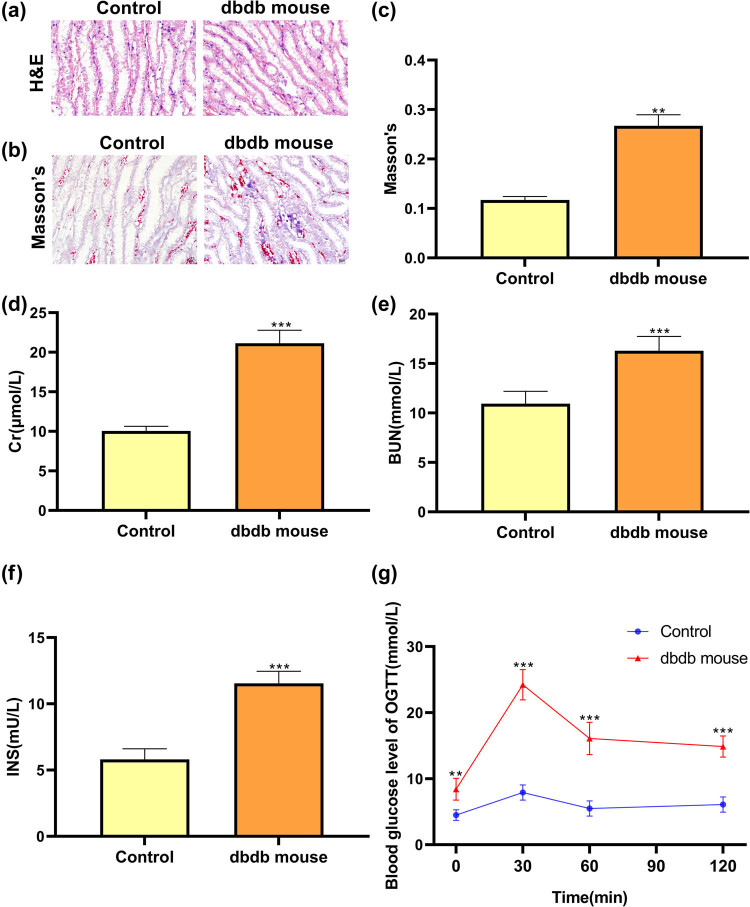
Detect the characteristics of db/db mice with spontaneous type 2 diabetes mellitus. Representative images of tissue stained with (a) H&E, (b) and (c) Masson’s are listed. Bar, 20 μm. (d) Cr (e) BUN levels were determined in the control and db/db mice. The result of (f) insuin (INS), (g) OGTT. **p < 0.01; ***p < 0.001.

Immunohistochemical and immunofluorescence results showed that ADAR1 in the proximal renal tubular cells of mice in the normal control group was mainly expressed in the cytoplasm and that the expression of ADAR1 in the cytoplasm of cells in the mice in the model group was significantly reduced. The expression levels of P-PI3K, p-AKT, and p-mTOR in the proximal renal tubular cells of mice in the model group were significantly higher than those in the proximal renal tubular cells of mice in the normal control group (Figure 1e–i, Figure A2). Western blot results indicated that the expression of ADAR1 in the model group was significantly decreased and the expression levels of P-PI3K, p-AKT, and p-mTOR in the model group were significantly higher than those in the normal control group (Figure 1j–n). The above experiments revealed the expression characteristics of ADAR1, PI3K, Akt, and mTOR in proximal renal tubular cells of db/db mice.

### 3.2 ADAR1 regulates SOD and MDA

**Figure 2:**
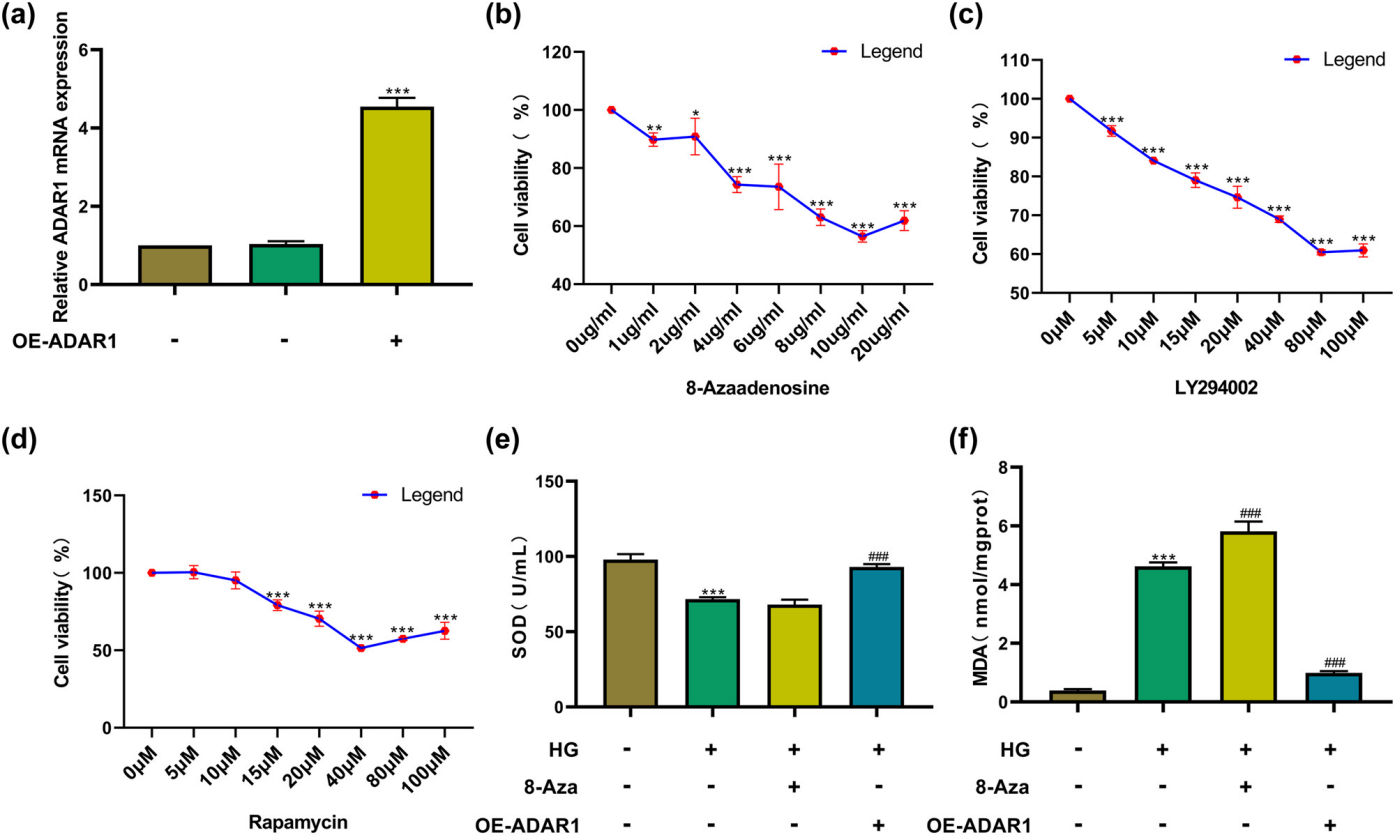
ADAR1 regulates SOD and MDA. (a) ADAR1-overexpression plasmids regulated ADAR1 mRNA expression level. The effects of different concentrations of (b) ADAR1 inhibitor 8-azaadenosine, (c) PI3K inhibitor LY294002, and (d) mTOR inhibitor rapamycin on the proliferation of HK-2 cells were screened by CCK8. (e) and (f) Detect SOD activity and MDA content under high-glucose conditions, ADAR1 inhibitor 8-azaadenosine, and ADAR1-overexpression. ***p < 0.001.

ADAR1-overexpression plasmids was constructed, RT-PCR results indicated that the ADAR1-overexpression plasmid upregulated ADAR1 mRNA expression (Figure 2a). To determine the optimal concentration of 8-azaadenosine, CCK-8 assay was performed to examine the cell viability of HK-2 cells treated with a series of concentrations of 8-azaadenosine. Our data showed that when the concentration of 8-azaadenosine reached 2 μM, distinct cytotoxicity appeared in HK-2 cells. Hence, 2 μM 8-azaadenosine was chosen as the optimal concentration (Figure 2b). CCK-8 assays showed that the ADAR1 inhibitor 8-azaadenosine (2 μM), PI3K inhibitor LY294002 (10 μM), and mTOR inhibitor rapamycin (10 μM) affected cell proliferation in a dose-dependent manner (Figure 2b–d). In the context of a high-glucose milieu, our findings unveiled a substantial decline in SOD activity accompanied by a significant rise in MDA content within HK-2 cells, indicative of heightened oxidative stress. After ADAR1 overexpression, SOD activity cells significantly increased, and MDA content significantly decreased under high-glucose conditions. However, the application of 8-azaadenosine showed a trend opposite to that of ADAR1 overexpression (Figure 2e and f). In conclusion, these data underscore the pivotal role of ADAR1 in modulating oxidative stress responses.

### 3.3 ADAR1 affects ER stress

**Figure 3:**
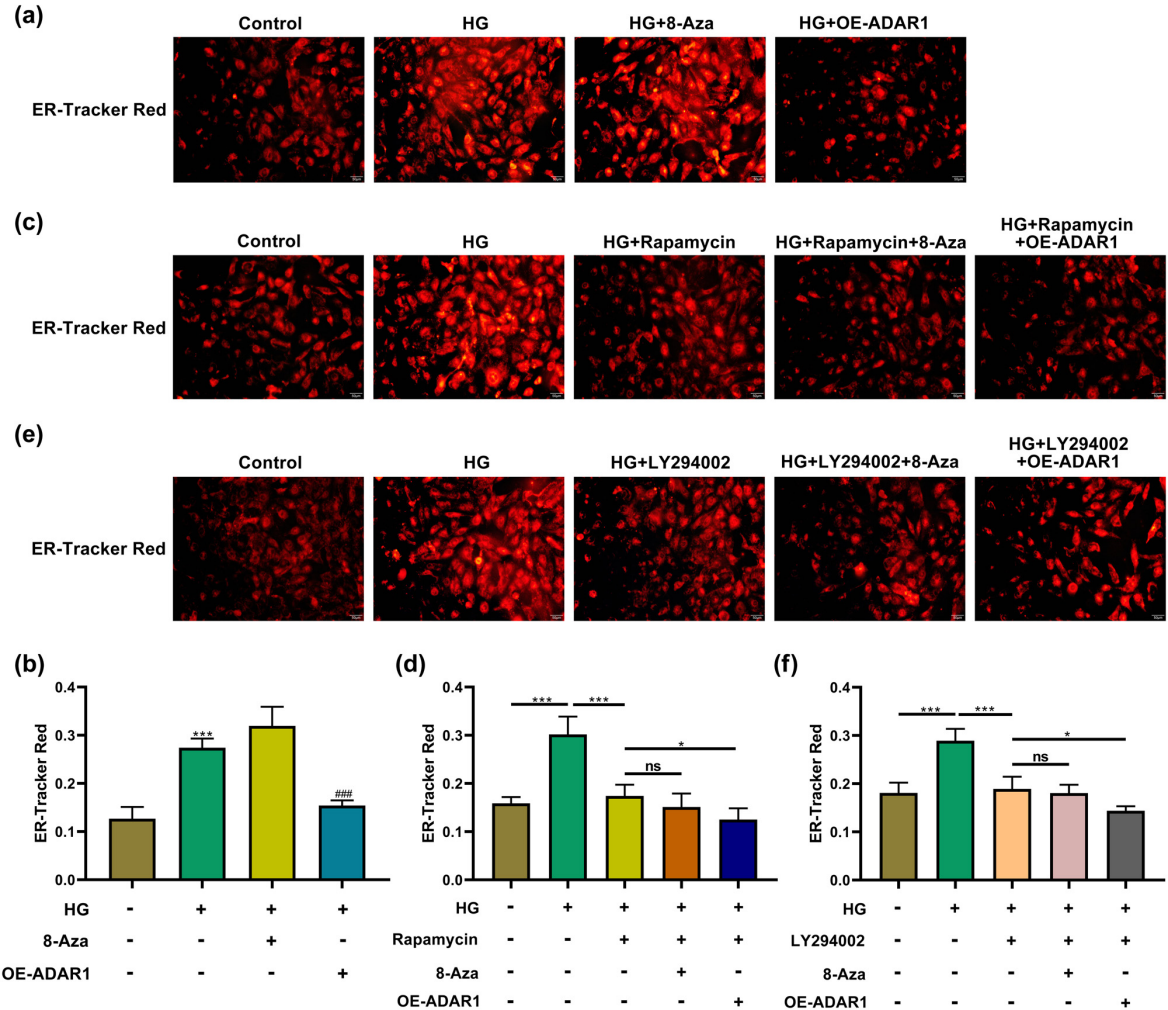
ADAR1 affects endoplasmic reticulum stress (a) and (b) ER-tracker dye detected effect of ADAR1 on the intensity of endoplasmic reticulum stress fluorescence probe under the high-glucose environment. (c) and (d) ER-tracker dye detected effect of ADAR1 on the intensity of endoplasmic reticulum stress fluorescence probe under high-glucose environment and rapamycin treatment. (e) and (f) ER-tracker dye detected effect of ADAR1 on the intensity of endoplasmic reticulum stress fluorescence probe under high-glucose environment and LY294002 treatment. Bar, 50 μm. *p < 0.05; ***p < 0.001.

Under a high-glucose environment, ADAR1 overexpression reduced the intensity of the ER fluorescent, and 8-azaadenosine enhanced the intensity of ER fluorescent, indicating high-glucose-induced ER stress (Figure 3a and b). Rapamycin reduced the intensity of ER-Tracker Red staining, indicating the amelioration of high-glucose-induced ER stress. The intensity of the ER fluorescent decreased more significantly after ADAR1overexpression, with no significant increase after ADAR1 inhibition (Figure 3c and d). Additionally, after treatment with LY294002, the expression pattern was basically the same as that after treatment with rapamycin (Figure 3e and f). ADAR1 may be closely related to the PI3K/mTOR signaling pathway in the mechanism underlying the regulation of ER stress.

### 3.4 Effect of ADAR1 on cell apoptosis and proliferation

**Figure 4:**
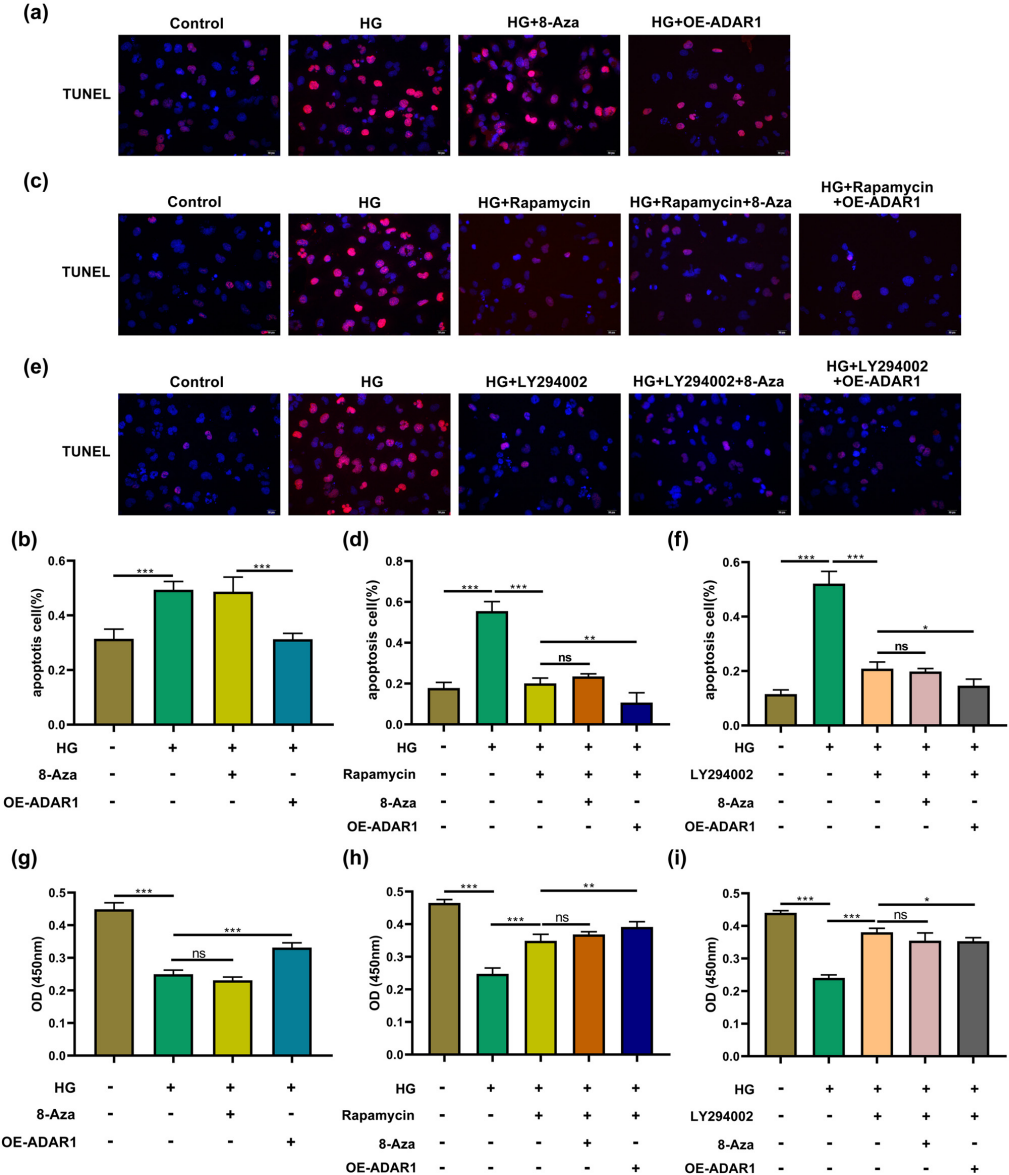
ADAR1 affects cell apoptosis (a) and (b) TUNEL staining assessed the effect of ADAR1 on the ratio of red fluorescent-labeled cells under the high-glucose environment. (c) and (d) TUNEL staining assessed the effect of ADAR1 on the ratio of red fluorescent-labeled cells under high-glucose environment and rapamycin treatment. (e) and (f) TUNEL staining assessed the effect of ADAR1 on the ratio of red fluorescent-labeled cells under high-glucose environment and LY294002 treatment. Bar, 20 μm. (g) CCK8 assays detected cell proliferation ability of HK-2 cells with HG, ADAR1 inhibitor 8-azaadenosine, and ADAR1 overexpression. (h) CCK8 assays detected cell proliferation ability of HK-2 cells with HG, ADAR1 inhibitor 8-azaadenosine, ADAR1 overexpression, and rapamycin. (i) CCK8 assays detected cell proliferation ability of HK-2 cells with HG, ADAR1 inhibitor 8-azaadenosine, ADAR1 overexpression, and LY294002. *p < 0.05; **p < 0.01; ***p < 0.001.

TUNEL assay results indicated that ADAR1 overexpression reduced the ratio of red fluorescently labeled cells compared with the HG group (Figure 4a and b). Rapamycin reduced the ratio of red fluorescently labeled high-glucose-induced cells, and on this basis, the ratio of red fluorescently labeled cells also decreased after ADAR1 overexpression, but there was no significant difference compared to 8-azaadenosine (Figure 4c and d). Similar profiles emerged upon exposure to LY294002 (Figure 4e and f). Cell proliferation assay showed ADAR1 overexpression enhanced cell proliferation compared with the HG group (Figure 4g). In a high-glucose environment, rapamycin and LY294002 enhanced cell proliferation, and on this basis, cell proliferation was enhanced after ADAR1 overexpression, but there was no significant difference compared to 8-azaadenosine. ADAR1 may be closely related to the PI3K/mTOR signaling pathway in the mechanism underlying the regulation of the cell apoptosis and proliferation.

### 3.5 Effect of ADAR1 regulation on the PI3K/AKT/mTOR signaling pathway

**Figure 5:**
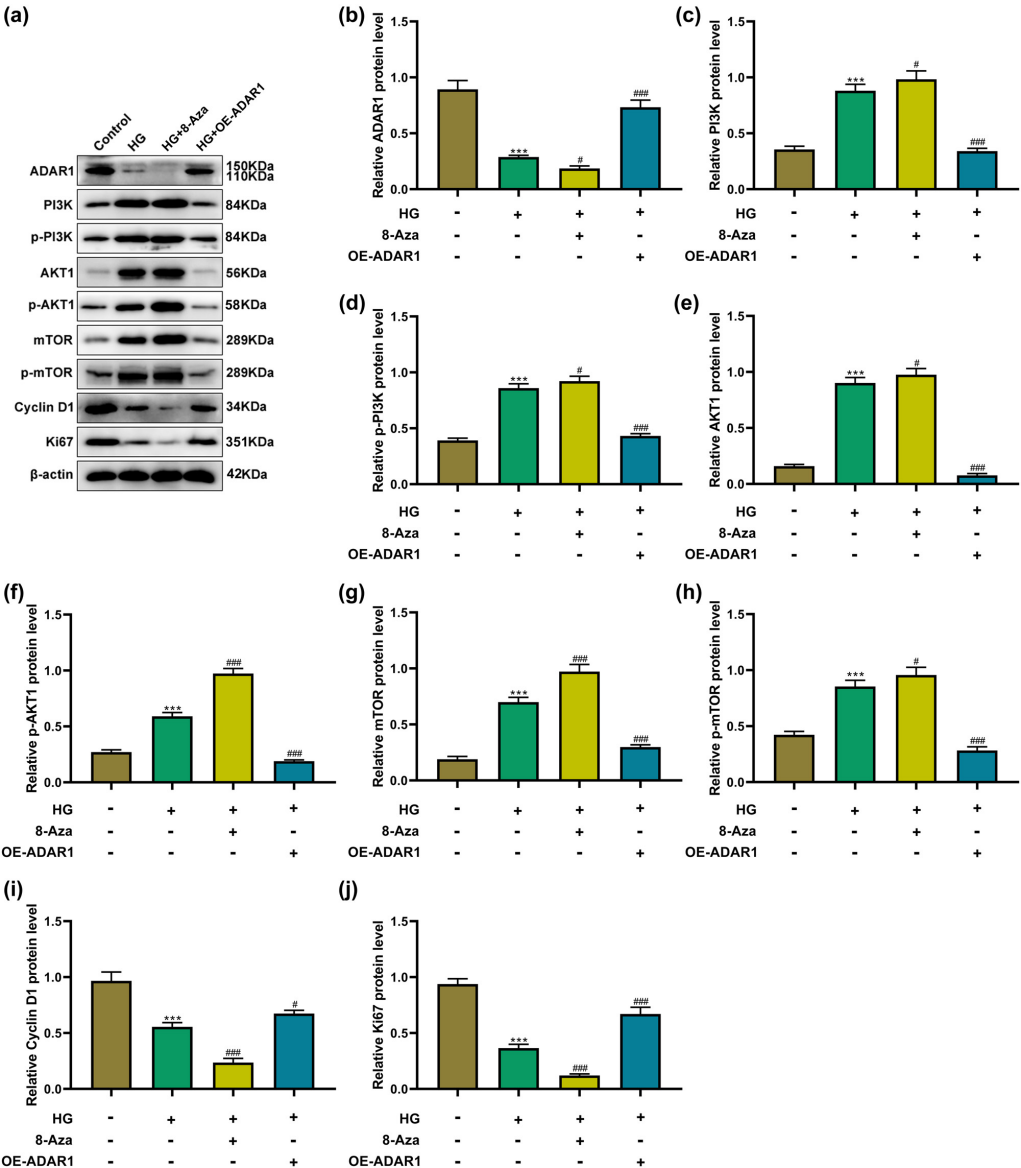
ADAR1 affects PI3K/AKT/mTOR signaling pathway under the high-glucose environment. (a)–(j) Expressions of (b) ADAR1, (c) PI3K, (d) p-PI3K, (e) AKT1, (f) p-AKT1, (g) mTOR, (h) p-mTOR, (i) cyclin D1, and (j) Ki67 were measured by western blot in HK-2 cells or ADAR1-overexpressed HK-2 cells under the high-glucose environment. ***p < 0.001.

Compared with the control group, the expression levels of ADAR1, Ki-67, and cyclin D1 in the high-glucose group were significantly lower. Additionally, the protein expression levels of phosphorylated p-PI3K/p-AKT/p-mTOR and non-phosphorylated PI3K/AKT/mTOR increased to varying degrees. Under high-glucose conditions, ADAR1 overexpression significantly upregulated ADAR1, Ki-67, and cyclin D1 expression and decreased the protein expression of phosphorylated p-PI3K/p-AKT/p-mTOR and non-phosphorylated PI3K/AKT/mTOR. Interestingly, 8-azaadenosine enhanced the effect of high-glucose factors under high-glucose conditions (Figure 5).

**Figure 6:**
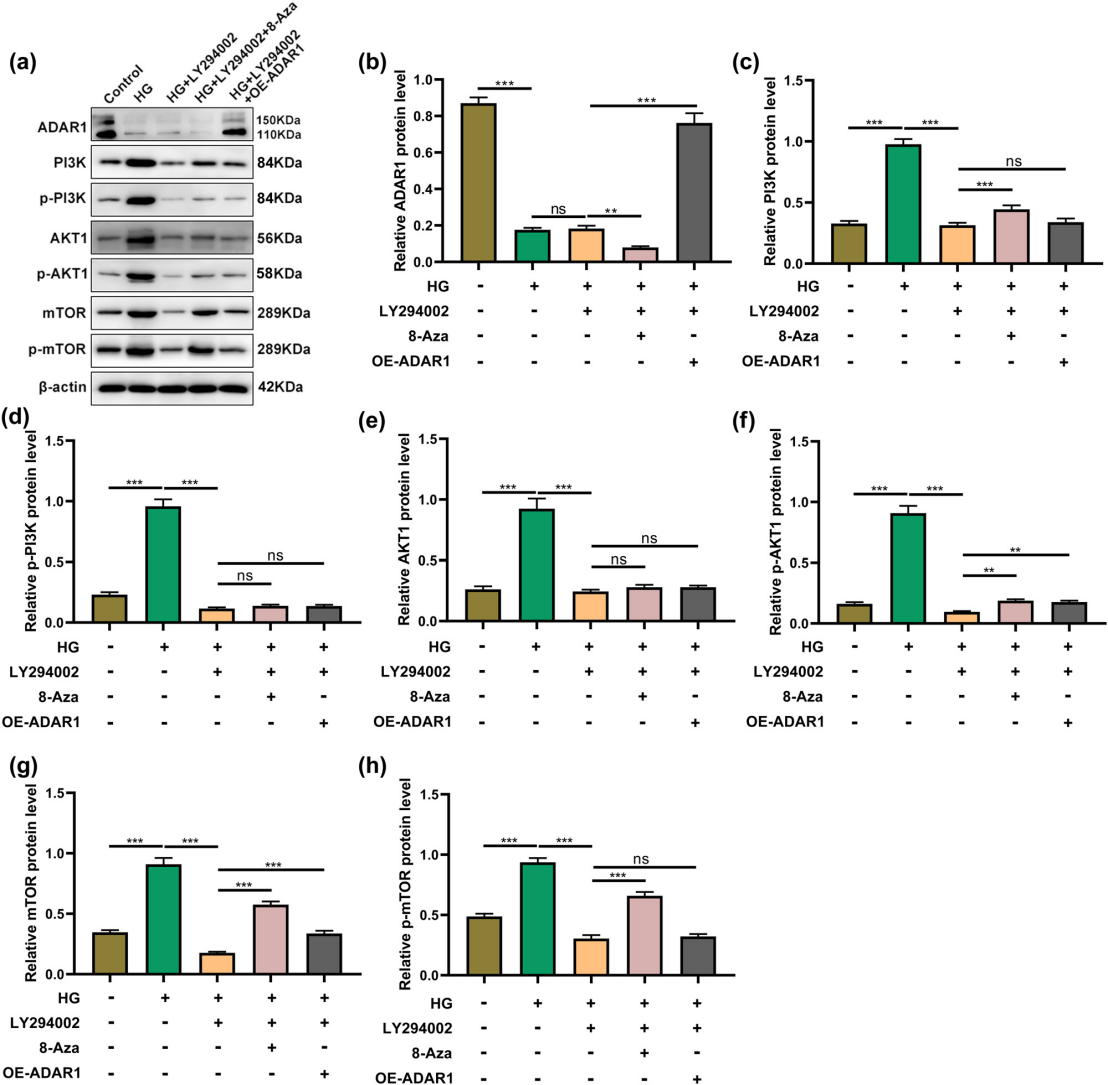
ADAR1 affects PI3K/AKT/mTOR signaling pathway under high-glucose environment and LY294002 treatment. (a)–(h) Expressions of (b) ADAR1 (c) PI3K, (d) p-PI3K, (e) AKT1, (f) p-AKT1, (g) mTOR, and (h) p-mTOR were measured by western blot in HK-2 cells or ADAR1-overexpressed HK-2 cells under high-glucose environment and LY294002 treatment. **p < 0.01; ***p < 0.001.

LY294002 significantly inhibited high-glucose-induced PI3K/AKT/mTOR protein expression. The expression levels of AKT and p-PI3K were not significantly different in cells treated with LY294002 combined with 8-azaadenosine and cells treated with LY294002 alone. The expression levels of p-mTOR, p- PI3K, and PI3K/AKT were not significantly different in cells treated with LY294002 combined with ADAR1 overexpression and cells treated with LY294002 alone (Figure 6).

**Figure 7:**
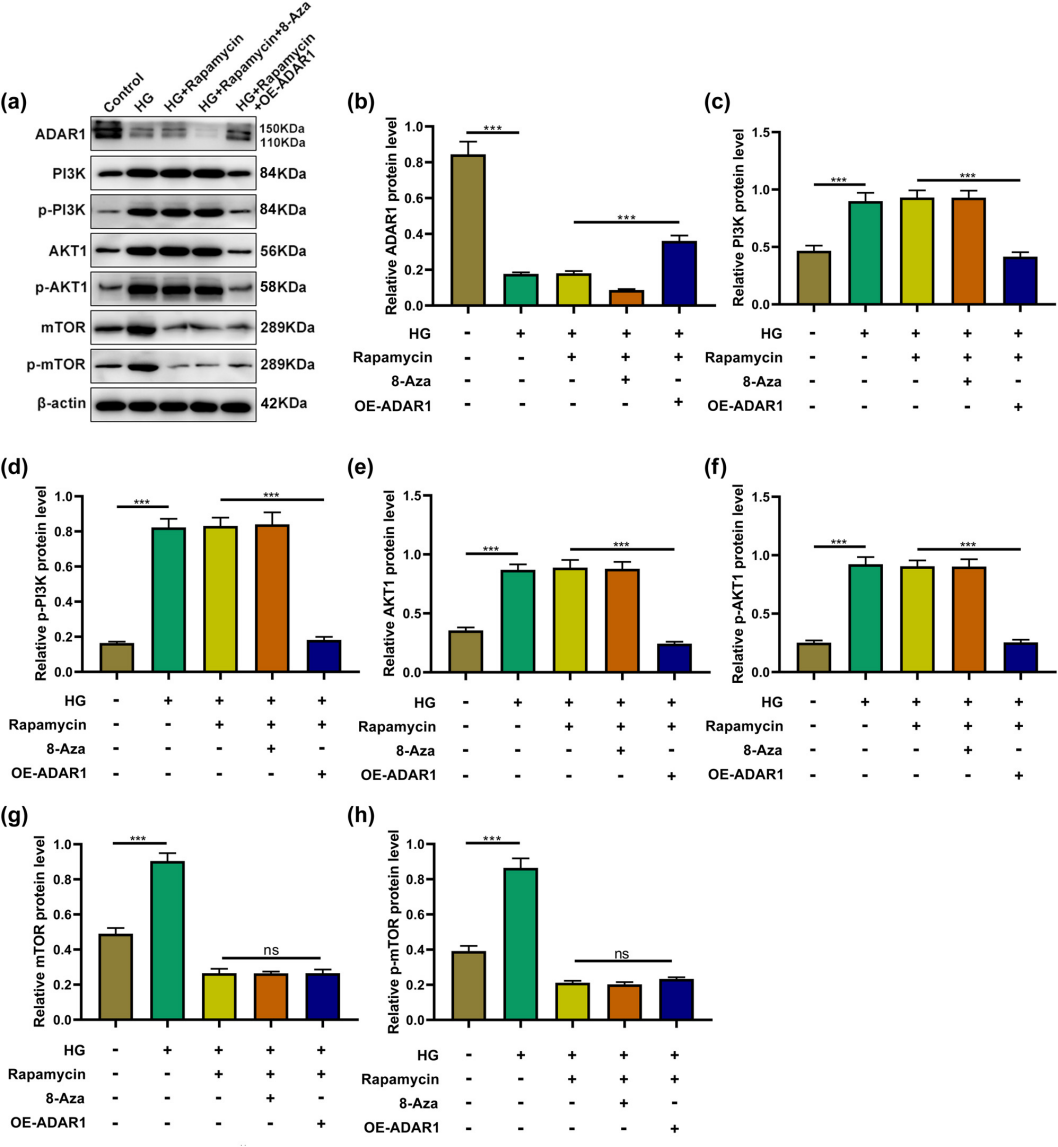
ADAR1 affects PI3K/AKT/mTOR signaling pathway under high-glucose environment and rapamycin treatment. (a)–(h) Expressions of (a) ADAR1 (c) PI3K, (d) p-PI3K, (e) AKT1, (f) p-AKT1, (g) mTOR, and (h) p-mTOR were measured by western blot in HK-2 cells or ADAR1-overexpressed HK-2 cells under high-glucose environment and rapamycin treatment. ***p < 0.001.

Rapamycin significantly inhibited high-glucose-induced mTOR and p-mTOR expression. In cells treated with rapamycin combined with 8-azaadenosine, there was no significant change in PI3K/AKT expression compared to applying rapamycin alone. Additionally, under high-glucose conditions, in cells treated with rapamycin and ADAR1 overexpression, all protein expression of phosphorylated and non-phosphorylated PI3K/AKT decreased; however, there was no significant change in mTOR and p-mTOR (Figure 7). The results further indicated that ADAR1 ameliorated high-glucose-induced oxidative stress and ER stress in HK-2 cells through the PI3K/AKT/mTOR signaling pathway.

## 4 Discussion

During the pathogenesis of DKD, proximal renal tubular cells undergo disturbances in their energy metabolism and aerobic processes as a consequence of hyperglycemic conditions, being prone to apoptosis, oxidative stress, and ER stress. These ultimately contribute to renal fibrosis and a decline in renal function [20]. Persistent hyperglycemic stress induces phenotypic transformation in proximal tubular cells, triggering the release of inflammatory-related factors and growth-promoting factors. This vicious cycle aggravates amplify hyperglycemia stimulation, expedites the apoptosis process in renal tubular cells, and impedes their regenerative and reparative capacities [21]. Previous studies identified ADAR1 as a novel target for cancer immunotherapy. As scholarly pursuits have progressed, the functional repertoire of ADAR1 has expanded beyond its recognized involvement in oncogenesis. ADAR1 was also found to prevent cell necrosis by inhibiting the accumulation of endogenous Z-RNA [22]. In aged cells and tissues, autophagy results in the downregulation of ADAR1, which facilitates the process of cellular senescence. Furthermore, ADAR1 knockdown has been demonstrated to induce cellular aging. Conversely, ADAR1 overexpression effectively impedes the progression of cellular aging [23]. The primary objective of our study was to delineate the expression characteristics of ADAR1 during the progression of DKD and to elucidate its functional mechanism in the context of high-glucose-induced HK-2 injury. We first characterized ADAR1 expression in proximal renal tubular cells using spontaneous type 2 diabetic mice. Second, examined the effect of ADAR1 on HK-2 cell proliferation, oxidative stress, and ER stress under a high-glucose environment. Finally, we explored the potential relationship between ADAR1 and the PI3K/AKT/mTOR signaling pathway. This study described the expression characteristics of ADAR1 in proximal renal tubular cells of diabetic db/db mice and elucidated the underlying mechanism by which ADAR1 alleviates high-glucose-induced HK-2 cell injury. Collectively, these findings revealed that ADAR1 may be a potential key factor in slowing the progression of DKD.

ADAR1 plays an essential role in maintaining physiological homeostasis, such as tissue cell proliferation and apoptosis [24]. The complete loss of ADAR1 activity is embryonically lethal, and ADAR1 is recognized as a negative regulator of ZBP1-mediated apoptosis [25]. Studies have also shown that the pathology effects caused by ADAR1 ZBD mutation are driven by the activation of ZBP1. Notably, the ablation of ZBP1 can effectively mitigate the pathological manifestations caused by ADAR1 modifications. However, it does not fully revert the inherent inflammatory response triggered by these ADAR1mutations [8]. ADAR1 alleviates high-fat diet-induced nonalcoholic fatty liver disease by inhibiting NLRP3 inflammasomes [26]. In this study, constructing an ADAR1-overexpression plasmid and applying an ADAR1 inhibitor (8-Aza) demonstrated that ADAR1 regulated the proliferation of HK-2 cells. Additionally, ADAR1 can regulate the levels of oxidative stress indicators such as SOD and MDA induced by high sugar, alleviating the cell apoptosis and oxidative stress induced by high sugar in HK-2 cells. Both oxidative stress and ER stress are acknowledged as integral components in the progression of DKD [27]. During the development of DKD, a sustained high-glucose stimulation results in transient ER stress in the early stage, which subsequently leads to ER stress-induced HK-2 cell damage during the later stages [28]. Interestingly, ADAR1 effectively alleviates the development of oxidative stress and ER stress, thereby underscoring its pivotal role in the progression of DKD.

In cancer research, ADAR1 has been implicated in the stabilization of the oncogene c-Myc via the AKT signaling pathway, a mechanism that fosters drug resistance in pancreatic cancer cells, specifically against BET inhibitors [29]. Knockdown of ADAR1 expression inhibits thyroid tumor proliferation and invasiveness [30]. ADAR1 emerges as a compelling biomarker for risk assessment in predicting recurrence of colorectal cancer in the remnant liver following surgical resection of liver metastases [31]. Beyond its oncological implications, the absence of the ADAR1 p150 isoform is implicated in the facilitation of autoinflammatory responses [32]. PI3K initiates a cascade of events by modifying the protein structure of AKT, subsequently activating it. This activation, in turn, orchestrates the phosphorylation-dependent regulation of proteins integral to the apoptotic machinery, thereby regulating cell proliferation, differentiation, apoptosis, and changes in the migration phenotype [33]. In the intricate cascade of apoptosis, mTOR, functioning as a downstream target of PI3K/Akt, also plays an important key role, especially in the progression of DKD [16]. In this study, we employed inhibitors of PI3K and mTOR to elucidate the mechanism underlying ADAR1’s protective effects on HK-2 cells. Although there have been related studies, the role of PI3K/AKT/mTOR signaling has not been investigated. To delve deeper into the connection between ADAR1’s impact on HG-induced HK-2 cells and the PI3K/AKT/mTOR pathway, our findings revealed elevated expression of PI3K, AKT, and mTOR in both diabetic mice and *in vitro* HG-stressed HK-2 cells. Notably, ADAR1 overexpression led to a substantial decrease in these proteins’ levels; conversely, inhibition of ADAR1 resulted in an increase. Furthermore, LY294002 can block the action of ADAR1, while rapamycin has no significant effect on the regulation of ADAR1. Therefore, we hypothesize that the PI3K/AKT/mTOR signaling pathway is activated in HG-induced HK-2 cells, and the upregulation of ADAR1 reduces the PI3K/AKT/mTOR signaling pathway and improves cell damage by reducing ER stress. Interestingly, 8-Aza upregulated mTOR protein expression after PI3K inhibition, and there was no significant change in mTOR expression when 8-Aza was applied together with the mTOR inhibitor rapamycin. Therefore, we believe that ADAR1 plays a role in the regulation of PI3K/AKT/mTOR pathway through multiple mechanisms, and there may be other crosstalk pathways. These hypotheses will be tested in future studies.

The two subtypes of ADAR1, p110 and p150, exhibit distinct expression across different cell types. In some cancer cell studies, the expression of ADAR1was significantly higher in the nucleus than in the cytoplasm, with predominant expression of the p110 subtype [34]. This study demonstrated a significant reduction in ADAR1 expression in the db/db mouse model and the high-glucose-induced HK-2 cell model. However, in this study, we did not delve into the expression differences and possible functional specificity of the p110 and p150 subtypes. Our study primarily focused on the expression of ADAR1 in the proximal tubules of DKD. Although we have not conducted an in-depth exploration and verification of the underlying mechanisms of renal tubular dysfunction, future research endeavors will aim to refine and expand the understanding within this field. Overall, ADAR1 upregulation can alleviate high-glucose-induced oxidative stress and ER stress in HK-2 cells by reducing PI3K/AKT/mTOR.

Taken together, these findings converge to highlight ADAR1 as a pivotal regulator with the potential to mitigate the progression of DKD. The results of *in vitro* and *in vivo* models showed that ADAR1 plays a protective role in proximal tubular cells under high-glucose conditions and could alleviate high-glucose-induced apoptosis, oxidative stress, and ER stress in HK-2 cells through PI3K/AKT/mTOR.
